# Hypercortisolism with coronary nonobstructive myocardial infarction and left ventricular noncompaction: a case report

**DOI:** 10.3389/fcvm.2026.1755172

**Published:** 2026-03-18

**Authors:** Yue Hu, Ruimin Chen, Zheng Wang, Yan Yang, Shufang Han

**Affiliations:** Department of Cardiology, The 960th Hospital of the Joint Logistic Support Force of PLA, Jinan, China

**Keywords:** case report, coronary artery spasm, hypercortisolism, left ventricular noncompaction, myocardial infarction with nonobstructive coronary arteries

## Abstract

This study reported a 40-year-old female patient admitted to hospital for acute myocardial infarction. Emergency coronary angiography showed no obvious vascular stenosis or occlusion, but radionuclide myocardial perfusion imaging suggested myocardial ischemia. Combined with the results of myocardial injury markers, the patient was diagnosed with MINOCA. In the process of further searching for the potential cause of MINOCA, she was found to have hypercortisolism with left adrenal cortex adenoma. Cardiac magnetic resonance performed during follow-up not only confirmed the anatomical location of the previous infarction but also suggested the concomitant presence of left ventricular noncompaction. This study provides a unique perspective to explore the complex interaction between endocrine dysfunction and myocardial injury, and also highlights the complexity of etiology and diagnostic challenges of MINOCA.

## 1 Introduction

Coronary atherosclerotic heart disease is one of the most common cardiovascular diseases, and its morbidity and mortality are increasing year by year. Acute myocardial infarction (AMI) is the most serious manifestation of coronary atherosclerotic heart disease and the leading cause of cardiovascular death (1). At present, with the rapid development of standardized drug therapy and coronary revascularization, the cure rate and long-term survival rate of patients with acute myocardial infarction have been significantly improved. In patients with acute myocardial infarction undergoing coronary angiography, most patients have significant coronary artery stenosis, but there are still about 6% of patients with coronary artery stenosis <50% (2). This special type of acute Myocardial infarction is Myocardial infarction with nonobstructive coronary arteries (MINOCA). Studies have shown that compared with patients with myocardial infarction of obstructive coronary artery (MICAD), MINOCA is characterized by a higher proportion of women and a younger age, and a relatively lower incidence of traditional cardiovascular risk factors (hypertension, dyslipidemia, diabetes, smoking, etc.). It has brought great challenges to the diagnosis of etiology and clinical treatment (3).

The degree of coronary artery stenosis in MINOCA patients is mild, but myocardial injury still occurs, which may be related to coronary artery spasm and microvascular dysfunction, coronary plaque destruction, spontaneous coronary thrombosis or embolism, spontaneous coronary artery dissection, and the mismatch between oxygen supply and oxygen supply (4). In addition to the above typical mechanisms, endocrine disease, as a systemic disease, can also be a rare driver of MINOCA. Hypercortisolism further aggravates the degree of coronary atherosclerosis and increases the incidence of acute myocardial infarction and mortality due to its long-term persistent high cortisol state (5). We report a case of special interest: a 40-year-old woman who was admitted to the hospital with an acute inferior ST-segment elevation myocardial infarction (STEMI) without substantial vessel stenosis or occlusion on coronary angiography. Further examination revealed hypercortisolism secondary to a left adrenocortical adenoma. The patient's cardiac magnetic resonance during follow-up not only confirmed the anatomical location of the previous infarction but also suggested the concomitant presence of left ventricular noncompaction (LVNC). This case provides a unique perspective on the complex interaction between endocrine dysfunction and myocardial injury, and also highlights the challenges in the diagnosis and treatment of MINOCA. In the diagnosis and treatment of such multi-factor diseases, the adoption of a holistic and multidisciplinary collaborative management mode is of great significance for the accurate diagnosis, individualized treatment and the improvement of the prognosis of patients.

## 2 Case presentation

The patient was a 40-year-old middle-aged woman who was admitted to the emergency room of our hospital due to persistent retrosternal pressure-like pain after emotional excitement, accompanied by chest tightness and sweating for half an hour. In the past, the patient underwent total thyroidectomy and neck lymph node dissection due to “right lobe thyroid cancer” in our hospital. After operation, he received thyroxine replacement therapy, and the condition was well controlled. The patient denied a recent history of fever or upper respiratory tract infection. He had no known history of allergies, tobacco or alcohol addiction. Her father and mother had hypertension and no other diseases.

**Figure 1:**
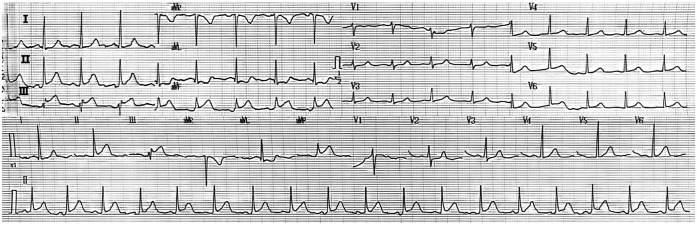
Electrocardiogram at presentation. (12-lead electrocardiogram showed sinus rhythm, ST-segment elevation in leads II, III, and aVF, and corresponding ST-segment depression in anterior leads, which was consistent with acute inferior STEMI.).

The initial physical examination of the emergency department doctor found that the patient was conscious and had an acute face. Vital signs were as follows: blood pressure 220/140 mmHg, heart rate 100 beats per minute, respiratory rate 20 breaths per minute, oxygen saturation 96%, and temperature 36.8 °C. Cardiopulmonary examination showed no obvious abnormalities and no peripheral edema. A 12-lead ECG was immediately performed on the patient, which revealed: sinus rhythm, ST-segment elevation of approximately 2–3 mm in inferior leads (II, III, and aVF), and corresponding ST-segment depression in anterior leads (V1-V3) (Figure 1). Given the typical electrocardiographic findings of acute inferior ST-segment elevation myocardial infarction, a treatment plan for STEMI was initiated. After on-site consultation and informed consent signed by the patient's family members, the patient was given aspirin 300 mg and imported clopidogrel bisulfate 300 mg, and the patient was urgently transported to the cardiac catheterization intervention room for coronary angiography.

### 2.1 Coronary angiography

**Figure 2:**
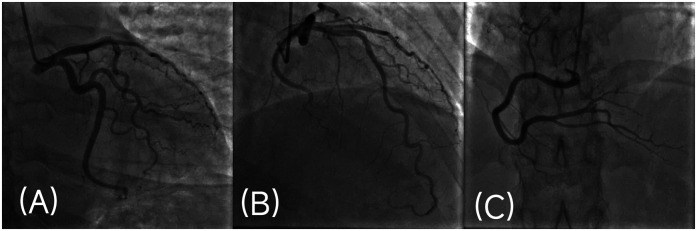
Coronary angiography image. **(A,B)** Left coronary angiography: angiography showed no obvious stenosis in LM, LAD and LCX, TIMI flow grade 3. **(C)** Right coronary angiography: angiography showed no obvious stenosis in RCA, TIMI flow grade 3.

Coronary angiography was performed through a right radial-artery approach. Angiography showed that the coronary artery was right-dominant, with normal opening, no obvious stenosis of the left main coronary artery (LM), smooth wall of the left anterior descending artery (LAD), left circumflex artery (LCX), and right coronary artery (RCA), and the distal blood flow was TIMI grade 3 (Figures 2A–C). No intravascular imaging examination such as coronary intravascular ultrasound or optical coherence tomography was performed during the acute operation.

### 2.2 Left ventriculography

In response to the patient’s discomfort symptoms such as chest pain and chest tightness after emotional excitement, we immediately performed an acute left ventricular angiography, which showed that the shape and motion of the left ventricle were good (Supplementary Figure S1), and there was no definite evidence of Takotsubo cardiomyopathy.

### 2.3 Laboratory examination and initial treatment

After surgery, the patient was admitted to the cardiology intensive care unit for further treatment and monitoring. In terms of laboratory examination, considering that the patient's onset was induced by emotional emotion, accompanied by rapid heart rate and elevated blood pressure, we adjusted the plasma epinephrine level for the patient on the day of onset: 36 pg/mL (normal 20–50 pg/mL), and the plasma norepinephrine level: The 24-hour urinary vanillylmandelic acid level was 10.29 mg/24 h (normal range: 0–12 mg/24 h). All the above results were within the normal range, and there was no clear evidence of pheochromocytoma. Outside the index visit, the patient was dynamically monitored for markers of myocardial injury, with an initial troponin I level of 0.011ug per liter (normal range, 0 to 0.0156), which peaked at 25 ug per liter approximately 15 h after admission. In addition, two biochemical tests revealed a low serum potassium level, ranging from 3.19 to 3.4 mmol per liter (normal, 3.5–5.5 mmol per liter). There were no obvious abnormalities in blood routine, coagulation routine, liver and kidney function, blood lipids and other routine laboratory tests. In terms of treatment, the patient was started on standard post-myocardial infarction therapy, including dual antiplatelet therapy (aspirin enteric-coated tablets 100 mg and imported clopidogrel bisulfate tablets 75 mg), statin (imported atorvastatin calcium tablets 20 mg), non-dihydropyridine calcium antagonist (imported diltiazem hydrochloride sustained release tablets 180 mg in two doses), and no dihydropyridine calcium antagonist (imported diltiazem hydrochloride sustained release tablets 180 mg in two doses). Oral potassium chloride sustained-release tablets were given to correct hypokalemia. Throughout the hospital stay, the patient was hemodynamically stable.

### 2.4 Diagnosis and etiology search of MINOCA

After the procedure, the patient underwent both resting and stress myocardial perfusion imaging (Supplementary Figures S2, S3). The results showed that the uptake of contrast agent in the apical and middle segments of the inferior posterior wall of the left ventricle was sparse, suggesting myocardial ischemia.

In combination with the findings on imaging and markers of myocardial injury and in the absence of definitive evidence of acute heart failure, pulmonary embolism, or Takotsubo cardiomyopathy, our initial diagnosis is “MINOCA”.

To identify the underlying cause of this patient's MINOCA, we reconducted a detailed history and physical examination. On careful examination, we noted a slightly round face and central obesity. This, combined with her recent history of elevated blood pressure, led us to consider the possibility of an underlying endocrine disorder. The absence of definitive evidence of pheochromocytoma made hypercorticolism more likely.

**Figure 3:**
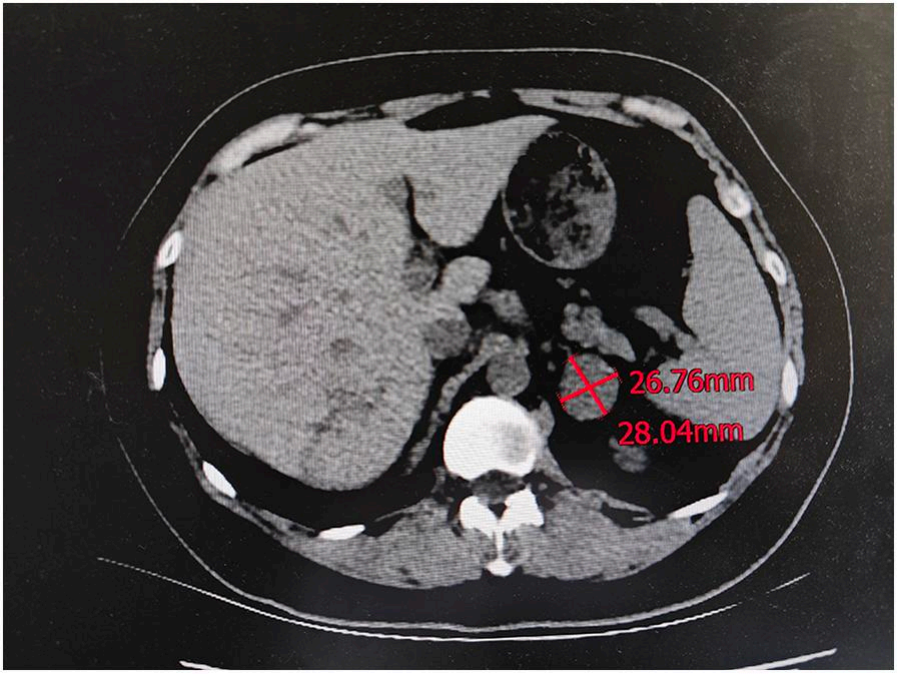
Computed tomography (CT) of the abdomen. CT of the abdomen shows a left adrenal mass measuring 2.8 cm by 2.6 cm.

Therefore, an evaluation for hypercortisolism was initiated. First, we measured patients’ serum cortisol levels at multiple time points to assess circadian rhythms: 00:00:406 nmol/L, 08:00:456 nmol/L, and 16:00:450 nmol/L, which indicated that the normal circadian variation in cortisol release had been lost. Second, the 24-hour urinary 17-hydroxycorticosteroid excretion was elevated to 18.45 mg/24 h (normal 2–10 mg/24 h), and the plasma adrenocorticotropic hormone (ACTH) level was suppressed to 5 pg/mL (normal 7.2–63.3pg/mL). All these results suggested that the cortisol source was ACTH-independent. To localize the source of the excess cortisol, computed tomography (CT) of the abdomen was performed. A scan revealed a well-defined, 2.8 cm × 2.6 cm, homogeneous soft-tissue mass in the left adrenal gland, suggestive of an adrenal adenoma (Figure 3). The appearance of the right adrenal gland was normal.

To make the diagnosis more rigorous, we performed a differential diagnosis of other related conditions and measured her 24-hour urinary 17-ketosteroid levels as follows: 9.63 mg/24 h (normal 6–25 mg/24 h). In addition, the patient's plasma levels of renin, angiotensin, aldosterone, thyroid hormone and sex hormone were all within the normal range, and cranial magnetic resonance imaging showed no obvious space-occupying lesions. Thyroid dysfunction, pheochromocytoma, and the presence of primary aldosteronism disease were excluded. Therefore, our initial diagnosis of the patient's hormonal abnormalities was hypercortisolism secondary to a left adrenal adenoma.

### 2.5 Discharge and follow-up

After 8 days of hospitalization, the patient's troponin I decreased to 0.239ug/L, and after evaluation of the patient's condition, we arranged for the patient to be discharged. In view of the presence of a left adrenal mass, we advised the patient that he still needed dynamic reexamination of the cortisol level and further surgery to confirm the final diagnosis.

### 2.6 Surgical intervention and pathological diagnosis

Two months after her initial presentation, the patient underwent successful laparoscopic left adrenal tumor resection in the urology department of our hospital. The surgical specimen included the left adrenal gland with a smooth surface, intact capsule, golden yellow oblate spherical nodule, approximately 3 cm in maximum diameter. Histopathological examination of the nodules showed that the nodules were consistent with adrenocortical adenoma. Further perfect the immunohistochemical: Inhibin-a(+)、Syn(+)、CD56(+)、S-100(-)、Melan-A(-)、CgA(-)、GATA-3(-)、Ki-67(2%+) (Supplementary Figure S4). After surgery, the patient's blood pressure and serum potassium level normalized without medication.

### 2.7 Cardiac magnetic resonance imaging

**Figure 4:**
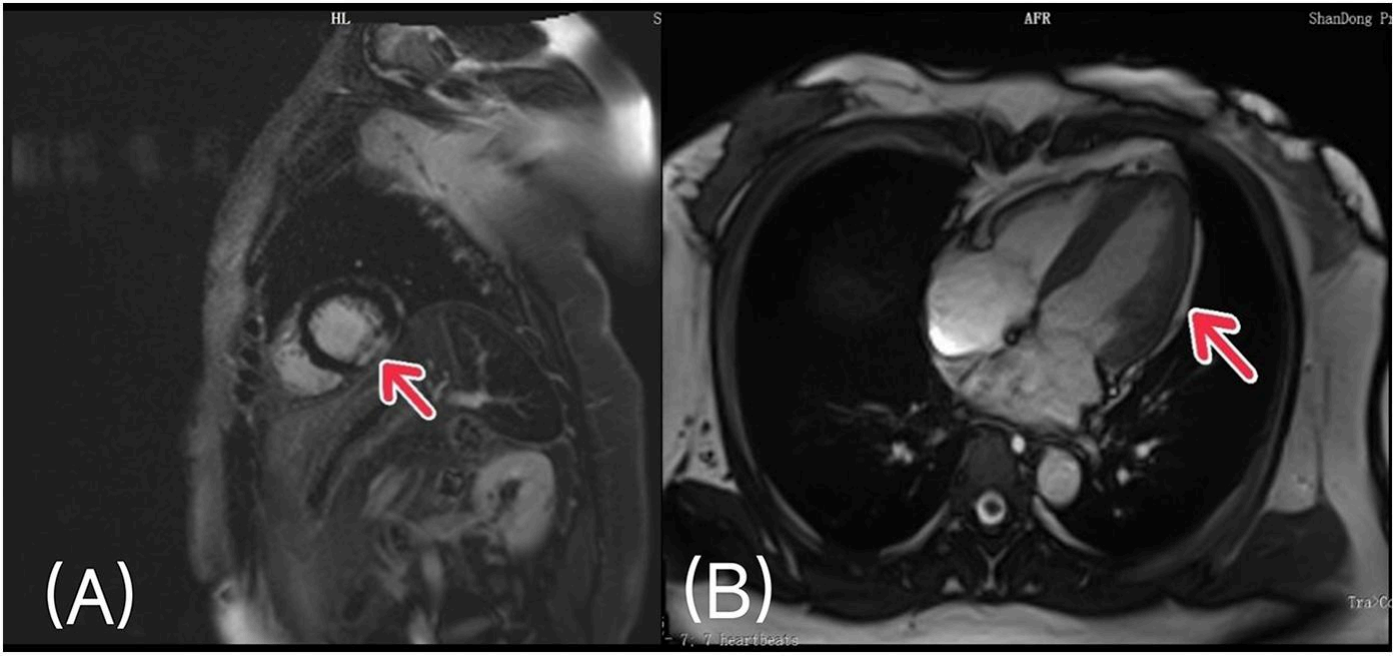
Cardiac magnetic resonance imaging: **(A)** patchy perfusion defects and delayed enhancement in the inferior wall of the middle and apical segments of the left ventricle. **(B)** The left ventricular free wall had increased muscle trabeculae and incomplete myocardial compaction.

Two months after adrenalectomy, the patient underwent cardiac magnetic resonance to further define the extent of myocardial injury and to assess global cardiac structure and function. Magnetic resonance imaging showed that the left ventricular ejection fraction was 58%. Myocardial perfusion scan showed patchy perfusion defects and delayed enhancement in the inferior wall of the middle and apical segments of the left ventricle, which was considered to be old myocardial infarction. In addition, MRI showed increased muscle trabeculae in the free wall of the left ventricle, which was considered as LVNC (Figures 4A,B).

The patient was followed up by the department of cardiology and endocrinology after discharge. During the one-year follow-up, the patient had no obvious discomfort symptoms, well controlled blood pressure, normal serum potassium level, and no cardiovascular events occurred. The postoperative patients’ serum cortisol levels were rechecked as follows: 00:00: 47 nmol/L, 08:00: 192 nmol/L, and 16:00: 85 nmol/L, all within the normal range. The postoperative patients’ cardiac color Doppler ultrasound examination showed that the sizes of all heart chambers were normal, and the ejection fraction was 58%.

## 3 Discussion

MINOCA patients met the clinical criteria of acute myocardial infarction, and coronary angiography showed that all potential pathogenic vessels stenosis <50% (6). At present, according to the pathogenesis, it is divided into two categories: ischemic (plaque rupture or erosion with transient thrombosis, coronary artery spasm, spontaneous coronary artery dissection, etc.) and non-ischemic (myocarditis, Takotsubo cardiomyopathy) (7). Among them, coronary plaque rupture is the most common cause of MINOCA, and its clinical characteristics and treatment have been widely studied and reported (8). However, there are relatively few reports of MINOCA caused by coronary spasm, and its treatment and prognosis are very different from those of coronary obstructive myocardial infarction. Based on the clinical examination results of this patient, we believe that coronary artery spasm further leads to an imbalance state between decreased coronary blood flow and increased myocardial metabolic demand, which is the main cause of MINOCA.

Cortisol hormones serve as key regulatory and stress hormones that maintain homeostasis in the cardiovascular system at physiological concentrations. However, high cortisol loading state has been proved to be one of the risk factors for aggravating atherosclerosis (5) and inducing spontaneous coronary artery dissection (9). We propose that a high cortisol state may trigger coronary spasm through several molecular mechanisms: first, a high glucocorticoid load promotes endothelial dysfunction and makes NO bioavailability less available. Excessive glucocorticoid directly inhibits the expression of endothelial nitric oxide synthase (eNOS) and reduces the production of nitric oxide (NO) (10) At the same time, it increases the release of reactive oxygen species (ROS) through mitochondrial electron transport chain, NAD(P)H oxidase and other pathways, which causes oxidative stress and accelerates the degradation of NO, thereby weakening the ability of vasodilation and triggering spasm (11). Second, excessive glucocorticoids enhance vascular smooth-muscle contractile activity. Glucocorticoid can directly activate the contractile signal of vascular smooth muscle by up-regulating the production of vasoconstriction factors endothelin-1 (ET-1) and angiotensin Ⅱ(AngⅡ), and increasing the expression of angiotensin Ⅱ type 1 receptor (AT-1) In addition (12), it also promotes calcium influx and activation of the RhoA/ROK pathway, which significantly enhances the contractility of coronary smooth muscle and leads to spasm (13). Thirdly, glucocorticoids promote the formation of coronary plaque and vascular remodeling, and aggravate atherosclerosis. The abnormal vascular wall structure at the site of plaque will further increase the susceptibility to coronary artery spasm (14).

LVNC is a congenital cardiomyopathy caused by myocardial compaction arrest in the embryonic period, which is mostly autosomal dominant inheritance (15). LVNC is characterized by abnormally thick muscle trabecula and deep recess communicating with the ventricular cavity, which is associated with heart failure, arrhythmia, thromboembolism and other events (16). There is an embryological and genetic association between LVNC and coronary artery development; studies have shown that during embryonic development, the process of ventricular muscle densification occurs simultaneously with the formation of the coronary artery vascular system, and both share key developmental pathways and genetic regulatory mechanisms (17). Myocardial densification involves multiple complex pathways such as Wnt/PCP signaling and Notch signaling (18). Experimental studies in mouse models have confirmed that abnormal development of dense myocardium is often accompanied by developmental defects in the coronary arteries. Specifically, the Dll4-Notch1 signaling pathway not only induces trabecula formation but also coordinates the densification of the myocardium with the morphogenesis of the coronary arteries (19). Additionally, specific genetic variations provide relevant evidence that the absence of Ino80 chromatin remodeler in endothelial cells not only hinders myocardial densification but also leads to defects in coronary artery vascular formation (20). Therefore, we propose a hypothesis that the underlying genetic factors of LVNC may lead to developmental disorders of the coronary artery endothelium, which may result in susceptibility to coronary artery spasm and further promote the occurrence of MINOCA.

The treatment process of this patient illustrates the importance of multidisciplinary collaboration: from emergency coronary angiography during the acute phase of chest pain, to subsequent etiology exploration, endocrine evaluation, surgical decision making, and long-term cardiac management, every step is inseparable from the shared decision-making of multidisciplinary teams. Based on the treatment of this patient, we summarized some clinical treatment inspirations: 1. For MINOCA patients, the central role of CMR in the diagnosis of MINOCA cannot be ignored, which is of great significance for identifying the type and degree of myocardial injury, achieving accurate etiological stratification, and detecting potential cardiomyopathy in time. (2) For young MINOCA patients without evidence of coronary atherosclerosis, it is necessary to broaden the differential diagnosis and actively screen for systemic causes. In the future, a risk prediction model for endocrine screening in MINOCA patients can be further studied and developed, a prospective registry can be established, and the prevalence of endocrine diseases in MINOCA population can be systematically assessed to form the best long-term management strategy. (3) With the development of genomics and metabolomics, it is still worth exploring whether biomarkers can be used to identify patients with high risk of MINOCA earlier, so as to achieve more precise primary prevention.

## 4 Conclusions

This case reports a rare case of hypercortisolism with MINOCA as the initial manifestation and LVNC. Coronary spasm caused by high cortisol load was considered to be the main cause of MINOCA in this patient. Therefore, in-depth exploration of the potential systemic etiology of MINOCA and accurate etiological treatment can fundamentally reverse its pathological damage to the cardiovascular system, help to reduce the recurrence rate of cardiovascular events, and improve the long-term prognosis of patients.

## Data Availability

The original contributions presented in the study are included in the article/Supplementary Material, further inquiries can be directed to the corresponding author.
