# Supplementary material for: Hypercortisolism with coronary nonobstructive myocardial infarction and left ventricular noncompaction: a case report
Source: Front Cardiovasc Med. 2026 Mar 18;13:1755172. doi: 10.3389/fcvm.2026.1755172 (PMC13038617; doi:10.3389/fcvm.2026.1755172)
Supplement: Supplementary file 2 [file Datasheet2.pdf]

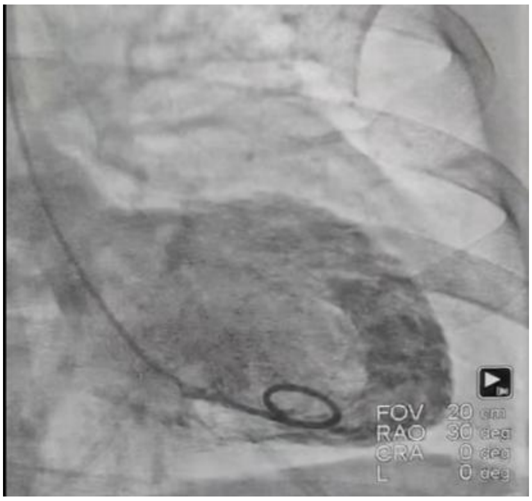


Supplementary Figure S1. Left Ventriculography: The structure and movement of the left ventricle were good.

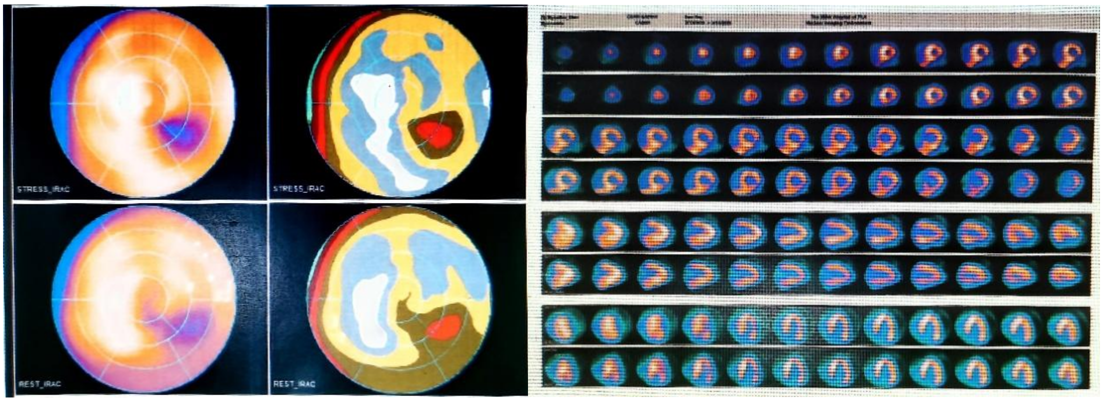

Supplementary Figure S2.S3. Radionuclide myocardial perfusion imaging: the uptake of contrast agent in the apical and middle segments of the inferior posterior wall of the left ventricle was sparse.

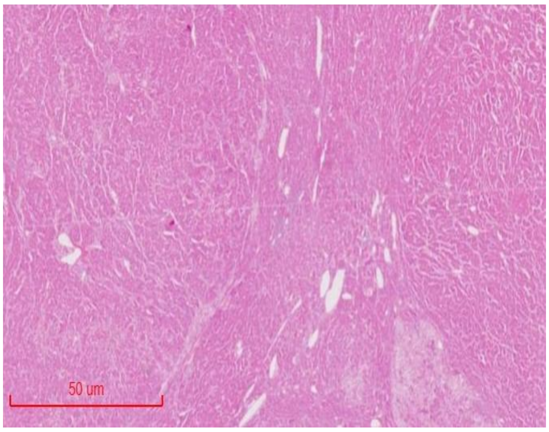


Supplementary Figure S4. Pathology of the Adrenal Nodule.
